# Evaluation of Acute Glial Fibrillary Acidic Protein and Ubiquitin C-Terminal Hydrolase-L1 Plasma Levels in Traumatic Brain Injury Patients with and without Intracranial Lesions

**DOI:** 10.1089/neur.2021.0048

**Published:** 2021-12-17

**Authors:** Peter Biberthaler, Ksenia Musaelyan, Sandro Krieg, Bernhard Meyer, Herbert Stimmer, Julian Zapf, Francesca von Matthey, Raj Chandran, Jaime A. Marino, Gangamani Beligere, Markus Hoffmann, Hongwei Zhang, Saul A. Datwyler, Beth McQuiston

**Affiliations:** ^1^ Department of Trauma Surgery, Technical University of Munich, Klinikum rechts der Isar, Munich, Germany.; ^2^ Core Diagnostics, Abbott Laboratories, Abbott Park, Illinois, USA.; ^3^ Department of Neurosurgery, Technical University of Munich, Klinikum rechts der Isar, Munich, Germany.; ^4^ Department of Radiology, Technical University of Munich, Klinikum rechts der Isar, Munich, Germany.; ^5^ Point of Care Division, Abbott Laboratories, Abbott Park, Illinois, USA.

**Keywords:** blood biomarkers, glial fibrillary acidic protein, point-of-care platform, S100 calcium-binding protein B, traumatic brain injury, ubiquitin C-terminal hydrolase L1

## Abstract

This pilot study aimed to evaluate the association of plasma ubiquitin C-terminal hydrolase-L1 (UCH-L1), glial fibrillary acidic protein (GFAP), and S100 calcium-binding protein B (S100B) with intracranial abnormalities visible on a computed tomography (CT) scan (CT positive) and injury severity in acute traumatic brain injury (TBI). For these purposes, a cohort of 109 adult TBI patients was recruited within 6 h from the injury event. A hyperacute subcohort of 20 patients who had their blood collected within 2 h from injury was analyzed separately for early acute biomarker levels. Levels of GFAP and UCH-L1 were analyzed using the prototype Abbott i-STAT™ TBI Plasma Test (Abbott Laboratories, Abbot Park, IL), alongside S100B measurement (Elecsys; Roche Diagnostics, Penzberg, Germany). In the hyperacute subcohort, GFAP and UCH-L1, but not S100B, levels were significantly higher in the CT-positive group compared to CT-negative patients. AUC values for differentiation between CT-positive and CT-negative patients were 0.97 for GFAP, 0.87 for UCH-L1, and 0.60 for S100B. Severity discrimination, defined by Glasgow Coma Scale (GCS) score, was then analyzed in the total patient cohort. Levels of all three biomarkers were significantly different between mild (GCS, 13–15) and moderate/severe (GCS, 3–12) injury groups. UCH-L1 showed the highest area under the curve value for severity discrimination (0.94), followed by GFAP (0.91) and S100B (0.83). These results support the clinical utility of GFAP and UCH-L1 as TBI biomarkers able to rule out CT-positive injury in acute TBI. Moreover, excellent differentiation of GFAP and UCH-L1 between mild and moderate/severe TBI groups affirms their close association with the underlying pathology.

## Introduction

Traumatic brain injury (TBI) is a highly prevalent condition in which incidence continues to rise across high-, middle-, and low-income countries.^1^ Currently, clinicians do not have many objective diagnostic tools at their disposal for the effective triage of TBI patients and thus rely on clinical assessment and the use of resource-demanding neuroimaging tools such as computed tomography (CT). The majority of TBI patients (up to 90%) are classified as mild (mTBI) based on a Glasgow Coma Scale (GCS) score of 13–15. Only a small proportion of mTBI patients tend to have any CT-positive abnormalities, yet use of the CT scan is very common in the evaluation of these patients.^2^ Wide use of head CT scans is associated with the potentially unnecessary risk of ionizing radiation exposure^3^ and leads to delays in the emergency department (ED) assessment related to scan and radiology staff availability.^4^ For these reasons, recent guideline reviews called for measures to reduce CT scan use in the management of TBI.^5^ At the same time, early detection of the small proportion of CT-positive patients among all TBI cases is a critical task of the ED team, given that these patients present an increased risk of developing neurosurgical conditions, such as intracranial hematomas, and early intervention significantly improves their prognosis and outcomes.

In view of this, a diagnostic test able to aid the selection of patients in need of a CT scan could be of great benefit for TBI management in the ED. Previously, evidence has supported the implementation of S100 calcium-binding protein B (S100B), an astrocytic protein, in Scandinavian countries and in some Western European hospitals. S100B was implemented as a blood biomarker able to aid in the rule-out of CT-positive abnormalities.^6,7^ Recently, two novel biomarker proteins, GFAP and UCH-L1, have been cleared for the rule-out of CT-positive abnormalities by the U.S. Food and Drug Administration.^8^ Research evidence available so far is showing considerable promise for their ability to aid in evaluation of CT-positive abnormalities, as well as for the selection of patients in need of magnetic resonance imaging (MRI) and treatment interventions.^9,10^ Multiple studies showed a correlation of these biomarkers with injury severity defined by GCS score.^11–13^ At the same time, studies that explored the timing of biomarker release after brain injury highlighted significant differences in the blood peak concentration times among biomarkers.^14^ As such, UCH-L1 was shown to peak immediately after injury whereas GFAP reaches its peak within the first 20–24 h.^15,16^ S100B is recommended for measurement within the first 6 h of injury,^6^ yet it has been proposed that its cerebral release occurs 24–30 h later.^17,18^ Such kinetic variations could explain existing inconsistencies among studies assessing biomarker performance at different intervals from the time of injury.

Thus, this study aimed to characterize acute biomarker response and its ability to rule out the presence of intracranial abnormalities within the very early post-traumatic period of 45 min to 6 h after the injury, when patients are most often assessed at the ED. Importantly, availability of a biomarker that can reflect both injury severity and underlying pathology would be highly beneficial for TBI clinical management, as well as for classification of patients in clinical trials where new targeted therapies are tested.^19,20^ Thus, in this report, we analyzed acute biomarker data derived from a prospective cohort of patients recruited for a Management of Traumatic Brain Injury Diagnosis (MIND) study. Dynamics of blood biomarker levels measured within the 2 h (hyperacute subcohort) and 6 h of injury (total cohort), as well their relationship with injury severity, has been investigated.

## Methods

### Study design and setting

TBI patients included in this analysis were enrolled at the ED of Klinikum rechts der Isar (Munich, Germany) for an Ethics Committee–approved observational, prospective MIND study. Klinikum rechts der Isar is a supraregional trauma center providing a maximum level of acute trauma care. Study recruitment was conducted jointly by clinical and research staff. Informed consent for participation in the study was obtained from patients or their legal representatives. Inclusion criteria for the study comprised age ≥18 years, new onset of neurological TBI symptoms after documented TBI, a blood draw taken within the first 6 h post-injury, and implementation of emergency neuroimaging testing per the site's accepted clinical practice.^21^

In mTBI cases, at least one or more of the following self-reported or eye-witnessed symptoms needed to be present for patient inclusion: Loss of consciousness (<30 min); post-traumatic amnesia (<24 h); alteration of consciousness; severe headache; and repeated vomiting (twice or more). Patients were excluded from the study if they refused to provide informed consent; were prisoners; had a known pre-existing neurological condition that could cause observed symptoms, or had a known recent history of TBI or seizures (≤1 year before the current ED presentation). In this analysis, a patient was assigned to the mTBI group if they had a GCS score of 13–15. All patients with more severe presentation (GCS, 3–12) were analyzed jointly as a moderate/severe TBI group, as has been done previously.^11^ The power analysis was not performed for this study design given that no target values for study outcomes were available from past literature.

### Sample collection and biomarker analysis

Blood sample collection occurred at the time of enrollment, and every effort was made to coordinate the blood draw with the blood draw ordered by a treating physician. Blood was collected into serum and ethylenediaminetetraacetic acid (EDTA) plasma tubes. Samples were then processed according to the manufacturers' instructions and stored at −70°C within 2 h of collection. Serum samples were tested at University College Dublin, and plasma GFAP and UCH-L1 samples were tested at Abbott Laboratories.

Sample analyses for GFAP, UCH-L1, and S100B were conducted by laboratory staff blinded to the clinical data. EDTA plasma was thawed in batches at room temperature and centrifuged at 10,000 relative centrifugal force (rcf) for 10 min before testing. GFAP and UCH-L1 concentrations were determined using prototype immunoassays evaluated simultaneously with a single cartridge on Abbott's i-STAT™ Alinity™ handheld point-of-care device (Abbott Laboratories, Abbott Park, IL) in duplicate. Sample analysis takes 15 min, and concentrations of both biomarkers are displayed on the analyser screen. These rapid tests use the sandwich enzyme-linked immunosorbent assay method with electrochemical detection of the resulting enzyme signal. The calibration range extended from 0 to 10,000 pg/mL for both GFAP and UCH-L1. The lower limit of quantification was 23 pg/mL for GFAP and 22 pg/mL for UCH-L1. The upper limit of quantification was 10,000 pg/mL for both GFAP and UCH-L1. Inter- and intrarun coefficients of variation were <10% for both assays.

Sample analysis for S100B was conducted by a single laboratory (University College Dublin), using an electrochemiluminescence immunoassay (Elecsys^®^ S100; Roche Diagnostics, Penzberg, Germany) on an automated Cobas^®^ system from Roche. Serum samples were thawed in batches at room temperature and centrifuged at 10,000 rcf for 10 min before testing. The measuring range for this assay is reported at 0.005–39.000 ng/mL.

### Computed tomography scan evaluation

All TBI patients included in this report had a CT scan assigned according to the local clinical practice guidelines. CT scans were analyzed by the radiologists of Klinikum rechts der Isar according to the local criteria. TBI patients were assigned to either CT-negative (no intracranial lesions present) or CT-positive (intracranial lesions present) groups according to CT scan results. CT-positive findings were defined as the presence of any of the following intracranial injuries: acute epidural hematoma; acute subdural hematoma; intraventricular hemorrhage; parenchymal hemorrhage or contusion; petechial hemorrhage/bland sheer injury; subarachnoid haemorrhage; brain oedema or herniation; and ventricular compression/trapping. Skull fractures were not evaluated as CT-positive intracranial injuries.

### Statistical analysis

To assess group differences between CT-positive and CT-negative patients, and between mild and moderate/severe groups, the Mann-Whitney U test was used, with *p* values ≤0.05 considered statistically significant.

Receiver operating characteristic (ROC) curves were constructed using a standard trapezoidal method, equivalent to the Mann-Whitney two-sample rank measure of association. All analyses were performed using SAS software (Version 9.4, copyright © 2020; SAS Institute Inc, Cary, NC).

## Results

### Characteristics of study subjects

This study included 109 TBI patients with samples collected within 6 h from injury (total cohort). Among those, 23 patients had CT visible abnormalities (CT positive) and 86 did not have any intracranial abnormalities visible on the CT scan (CT negative). Twenty patients from this cohort had their blood collected between 45 min and 2 h from the time of injury. These patients were included into a hyperacute subcohort, which was used for a separate hyperacute biomarker analysis. Demographic details for the total and hyperacute cohorts are provided in Table 1.

**Table 1.** Demographic Characteristics and Biomarker Analysis in the Study Cohort

	Total cohort: (blood draw 0–6 h from injury)	Hyperacute subcohort: (blood draw 0–2 h from injury)
Characteristics	N = 109	N = 20
Age, mean (SD) [range], years	59 (22) [18, 97]	61 (23) [25, 89]
Male sex, no. (%)	58 (53)	12 (60)
Race/ethnicity, no. (%)		
White	105 (96)	19 (95)
Asian	1 (1)	1 (5)
Other/unknown race	3 (3)	0 (0)
GCS score in study site, no. (%)		
3	5 (5)	2 (10)
12	2 (2)	1 (5)
13	3 (3)	0 (0)
14	6 (6)	2 (10)
15	93 (85)	15 (75)
Loss of consciousness, no. (%)		
Y	31 (28)	3 (15)
N	78 (71)	17 (85)
Head CT scan		
Traumatic injury on head CT, no. (%), (CT positive)	23 (21)	3 (15)
No traumatic injury on head CT, no. (%), (CT negative)	86 (79)	17 (85)
Rapid assay results		
Time from injury to blood draw, median, [range], (IQR) h	3 [0.8, 6.0] (2.3–4.2)	1.3 [0.8, 2.0] (0.9–1.8)
GFAP, median, [range], (IQR) pg/mL	73 [3, 20,026] (36, 186) [*n* = 109]	77 [8, 1546] (42, 219) [*n* = 20]
UCH-L1, median [range], (IQR) pg/mL	282 [46, 13,124] (152, 396) [*n* = 109]	496 [79, 13,124] (243, 615) [*n* = 20]
S100B, median [range], (IQR) ng/mL	0.15 [0.02, 7.50] (0.09, 0.26) [*n* = 109]	0.25 [0.05, 7.50] (0.16, 0.42) [*n* = 20]

SD, standard deviation; GCS, Glasgow Coma Scale; CT, computed tomography; IQR, interquartile range; GFAP, glial fibrillary acidic protein; UCH-L1, ubiquitin C-terminal hydrolase-L1; S100B, S100 calcium-binding protein B.

### Biomarker response in hyperacute subcohort

To understand the acute dynamics of biomarker response, we analyzed a hyperacute subcohort of patients who had their blood collected within 2 h of injury. In this subcohort of 20 patients with a mean age of 59 years, 17 did not have any intracranial injury visible on a CT scan (CT negative) and 3 had CT-visible abnormalities (CT positive). GFAP levels were significantly higher in the CT-positive group compared to the CT-negative group (*p* = 0.010). UCH-L1 was also significantly elevated in the CT-positive group compared to the CT-negative group (*p* = 0.010). There was no significant difference found in the concentrations of S100B between CT-negative and CT-positive groups (*p* = 0.458). For median, mean, and range of biomarker levels in CT-positive and CT-negative groups of the hyperacute subcohort, see Table 2.

**Table 2.** Protein Levels in Blood Samples of Hyperacute Subcohort Collected within 2 h from Injury

	Median (25th–75th percentile)	Mean (SD)	Range	p value (vs. CT-negative group)
GFAP, pg/mL				
CT negative	56 (37–114)	92 (88)	8–333	n/a
CT positive	833 (240–1546)	873 (654)	240–1546	0.010
UCH-L1, pg/mL				
CT negative	431 (190–551)	409 (227)	79–915	n/a
CT positive	2041 (662–13,124)	5276 (6832)	662–13,124	0.010
S100B, ng/mL				
CT negative	0.29 (0.161–0.407)	0.42 (0.469)	0.05–1.89	n/a
CT positive	0.17 (0.164–7.540)	2.62 (4.26)	0.16–7.54	0.458

CT, computed tomography; IQR, interquartile range; GFAP, glial fibrillary acidic protein; UCH-L1, ubiquitin C-terminal hydrolase-L1; S100B, S100 calcium-binding protein B; SD, standard deviation; n/a, not applicable.

**FIG. 1.**
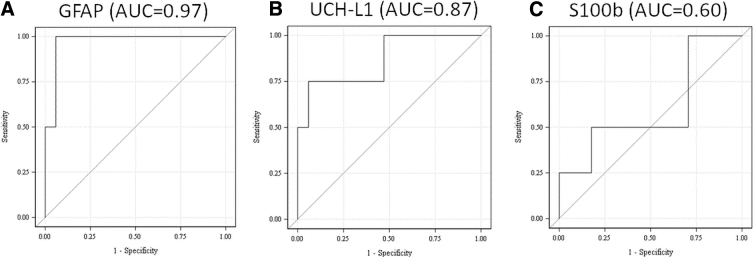
Receiver operating characteristic curve demonstrating diagnostic performance of plasma glial fibrillary acidic protein (GFAP) and ubiquitin C-terminal hydrolase L1 (UCH-L1), as well as serum S100B measured within 2 h of injury in distinguishing between TBI patients with and without CT-positive intracranial lesions. The area under the curve (AUC) shows that GFAP (**A**) and UCH-L1 (**B**) could distinguish between the two groups with high accuracy (AUC = 0.97 and AUC = 0.87 respectively), whereas S100B (**C**) demonstrated suboptimal performance (AUC = 0.60). CT, computed tomography; S100b, S100 calcium-binding protein B; TBI, traumatic brain injury.

To further explore biomarker ability to distinguish between CT-negative and CT-positive TBI patients, ROC curves were constructed along with 95% Wald confidence intervals (CIs). Area under the curve (AUC) results showed that GFAP could distinguish between the two patient populations with the highest AUC (AUC = 0.97; CI, 0.90, 1.00), followed closely by UCH-L1 (AUC = 0.87; CI, 0.63, 1.00). S100B showed a comparatively suboptimal performance in this analysis (AUC = 0.60; CI, 0.22, 0.98; see Fig. 1).

### Biomarkers and injury severity

**FIG. 2.**
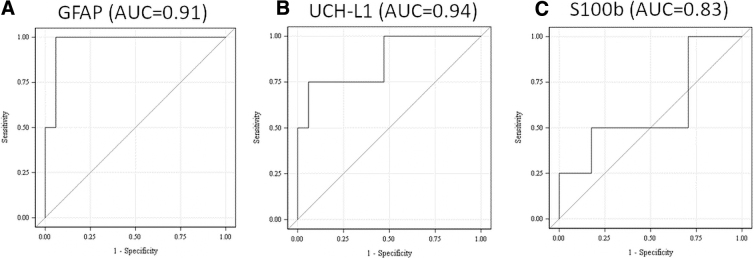
Receiver operating characteristic curve demonstrating diagnostic performance of (**A**) plasma glial fibrillary acidic protein (GFAP) and ubiquitin C-terminal hydrolase L1 (UCH-L1), as well as serum S100b for distinguishing between mild and moderate/severe TBI patients. The area under the curve (AUC) calculation shows that UCH-L1 (**B**) was the most accurate in distinguishing between the two groups (AUC = 0.94), followed closely by GFAP (AUC = 0.91; panel A) and S100B (AUC = 0.83; panel **C**). S100b, S100 calcium-binding protein B; TBI, traumatic brain injury.

To understand the relationship between blood biomarkers and injury severity, the ability of biomarkers to distinguish between mild versus moderate/severe TBI, as defined by GCS score, in the total cohort was analyzed. In the total cohort of 109 TBI patients, 7 subjects had a GCS score of 3–12 and thus were classified as TBI moderate/severe. A comparison of biomarker levels between mild and moderate/severe groups showed that all three biomarker levels were significantly elevated in the latter group (Mann-Whitney U; *p* ≤ 0.0001). For median, mean, and range of biomarker levels in mild and moderate/severe groups in the total cohort, see Table 3. ROC curve analysis showed that UCH-L1 was able to distinguish between the two groups of patients with an AUC of 0.94 (95% Wald CI, 0.862, 1.00). UCH-L1 was followed closely by GFAP with an AUC of 0.91 (95% Wald CI, 0.843, 0.982). S100B was able to distinguish between the two groups with an AUC of 0.83 (0.679, 0.988) (for ROC curves, see Fig. 2).

**Table 3.** Protein Levels in Mild and Moderate/Severe TBI Samples Collected within 6 h of Injury

	Median (25th–75th percentile)	Mean (SD)	Range	p value (vs mild TBI group)
GFAP, pg/mL				
Mild TBI	64 (33–138)	111 (134)	3–864	n/a
Moderate/severe TBI	764 (272–3776)	3381 (6145)	176–20,026	<0.0001
UCH-L1, pg/mL				
Mild TBI	237 (125–377)	282 (198)	46–1390	n/a
Moderate/severe TBI	705 (437–2162)	2556 (3981)	389–13,124	0.0001
S100B, ng/mL				
Mild TBI	0.14 (0.084–0.237)	0.21 (0.247)	0.02–1.89	n/a
Moderate/severe TBI	0.60 (0.253–1.570)	1.52 (2.297)	0.16–7.54	0.0001

TBI, traumatic brain injury; GFAP, glial fibrillary acidic protein; UCH-L1, ubiquitin C-terminal hydrolase-L1; S100B, S100 calcium-binding protein B; SD, standard deviation; n/a, not applicable.

## Discussion

This pilot study, for the first time, demonstrates the analysis of blood biomarkers GFAP and UCH-L1 in the hyperacute time frame of the first 2 h after a brain injury event in a small subcohort of patients, as well as within a wider window of the first 6 h post-injury in the total cohort. We show that GFAP and UCH-L1 can successfully rule out CT-positive abnormalities across the full spectrum of TBI severity within this time frame with a better ROC curve performance than that of S100B.

This study recruited 109 TBI patients with a GCS score of 3–15, who had their blood samples collected within 6 h from injury. Demographically, this cohort was characterized by an average age of 59 years and a predominance of mTBI cases (93%). Such characteristics reflect the reported epidemiology of TBI patients in high- and middle-income countries.^22,23^ Prevalence of CT-positive findings was 21% in the total cohort, which is somewhat higher than the prevalence reported in observational studies previously.^2^ This is likely attributable to the study design, given that indication for a CT scan was among the inclusion criteria to ensure that neuroimaging data were available for each case. Such a study design has been previously adopted among many large, multi-central studies, such as TRACK-TBI^24,25^ and CENTER-TBI.^13^

From the total cohort, we extracted a hyperacute subcohort of 20 patients who had their blood samples collected as early as 45 min and within 2 h from injury. This subcohort lent itself for the analysis of the very early dynamics of biomarker release post-injury. We showed that GFAP and UCH-L1 plasma levels were already significantly higher in CT-positive patients compared to the CT-negative group in this subcohort. S100B was also elevated in this group; however, some CT-negative patients also had elevated S100B levels, and hence no significant difference was found between the two groups. Lower AUC values for S100B observed in the hyperacute subcohort are in line with previous studies showing GFAP outperforming S100B in the detection of CT abnormalities.^25,26^ Although S100B has previously shown a high sensitivity to detect CT abnormalities, its specificity for intracranial lesion detection has been reported to be low.^7^ This could be related to the extracranial sources of the S100B protein and its elevation in orthopedic trauma previously described in the literature.^27–29^

To our knowledge, our study is one of the first to assess the ability of GFAP and UCH-L1 to detect CT abnormalities as early as within the first 2 h of injury. Most studies suggest that UCH-L1 levels are acutely elevated post-injury, and its levels peak within the first 8 h.^15,16^ Our data confirm this notion and suggest an even earlier peak, given that the range of 2-h elevation was higher than the range detected within 6 h. This observation is in line with some experimental studies, which found an increase in UCH-L1 plasma levels as early as 5 min after experimental injury in rodents.^30^ Interestingly, GFAP was also elevated within the first 2 h, in contrast with previous studies that show GFAP levels elevated from 4 h post-injury and peaking at ∼20 h.^15,16^ However, those few studies that looked at the diagnostic performance of this biomarker at earlier stages, within 4 and within 6 h post-injury, also detected high AUC values for detection of CT-positive abnormalities.^26,31^

High AUC values for GFAP and UCH-L1 in this analysis indicate that these biomarkers are suitable for CT rule-out in the acute setting, if available at the point of care, such as in the emergency room or on a sports field. However, it is important to acknowledge that the hyperacute subcohort included a small number of patients, given that patient recruitment within the first 2 h of trauma presents a challenge for clinical investigators. As in any TBI population, only a small number (*n* = 3, or 15%) of patients in this already limited group turned out to be CT positive. All of the CT-positive patients presented with GCS below 13 and were therefore assigned to a combined moderate/severe TBI group. Thus, the percentage of moderate/severe TBI patients was also higher in this subcohort compared to the total cohort, where only 7% of patients were moderate/severe. Although use of GCS for severity classification has been repeatedly called into question in recent years,^32,33^ the overlap between severity and CT findings produced a level of ambiguity in the interpretation of results. Nonetheless, we believe that these pilot data showing an increase of GFAP and UCH-L1 in the first 2 h from moderate/severe TBI contribute novel evidence and warrant further investigation of these biomarkers' kinetics and diagnostic performance in the first hours after brain injury.

Importantly, biomarker levels measured within 6 h from injury also distinguished between patients with mild and moderate/severe TBI in the total cohort of 109 patients. Moderate and severe TBI patients were combined into a single group as has been done previously.^11^ In our study, all three measured proteins showed good discrimination between the two severity groups. This finding is consistent with previous studies, which found all three biomarker levels correlating with GCS score.^13^ Similarly, GFAP^12^ and UCH-L1^11^ levels have been shown to differ among patients stratified by GCS severity. However, ours is the first study to report AUC values for biomarkers' discriminative ability between mild and moderate/severe TBI. Biomarker ability to distinguish between severity groups strengthens the evidence of its connection to the underlying pathology and potentially paves the way for its monitoring and prognostic use.

In our study, UCH-L1 showed the highest AUC value for injury severity discrimination within 6 h of injury, followed closely by GFAP. Such an excellent UCH-L1 performance could be attributed to the early timing of sample collection. Similarly, a previous study that also compared biomarker performance within 6 h post-injury found the highest discriminative ability of UCH-L1 for CT abnormalities compared to GFAP and S100B.^31^ Another factor that could have enhanced UCH-L1 performance in our study is the use of a highly sensitive laboratory assay designed for future clinical use. Given that UCH-L1 as well as GFAP testing is not yet conducted by hospital laboratories, research use-only assays may significantly vary in their precision and accuracy.^34,35^

S100B also showed good performance in severity discrimination. This finding is in line with previous studies that showed a S100B correlation with injury severity as detected by CT and MRI examinations.^36,37^ At the same time, it is important to consider that extracranial injury has been shown to significantly contribute to peripheral S100B levels.^27^ Hence, it is possible to speculate that S100B in the blood of patients with more severe injury, who are more likely to have sustained injuries to other tissues, comes from multiple sources not exclusive to the brain.

This pilot study is limited by the small sample size of subgroups used for analysis, such as CT-positive patients seen within 2 h from injury and patients with moderate/severe TBI. Further investigation in a larger cohort of TBI patients, particularly those with mild TBI recruited within the first hours post-injury, is needed to address this limitation.

## Conclusion

In this pilot study, hyperacute plasma GFAP and UCH-L1 levels were analyzed in a unique cohort of TBI patients recruited as early as within the first 2 h from injury. The preliminary analysis demonstrated good GFAP and UCH-L1 performance for detection of CT-positive abnormalities and stratification by injury severity. These promising data warrant further research into the early kinetics of these brain-injury biomarkers in larger cohorts of TBI patients with varied CT findings and injury severity.

## Notes

**Cite this article as:** Biberthaler, P, Musaelyan, K, Krieg, S, Meyer, B, Stimmer, H, Zapf, J, von Matthey, F, Chandran, R, Marino, JA, Beligere, G, Hoffmann, M., Zhang, H, Datwyler, SA, and McQuiston, B (2021) Evaluation of acute glial fibrillary acidic protein and ubiquitin C-terminal hydrolase-L1 plasma levels in traumatic brain injury patients with and without intracranial lesions. *Neurotrauma Reports* 2:1, 617–625, DOI:10.1089/neur.2021.0048.

## Abbreviations Used

AUC: area under the curve
CI: confidence interval
CT: computed tomography
ED: emergency department
EDTA: ethylenediaminetetraacetic acid
GCS: Glasgow Coma Scale
GFAP: glial fibrillary acidic protein
MRI: magnetic resonance imaging
mTBI: mild TBI
rcf: relative centrifugal force
ROC: receiver operating characteristic
S100B: S100 calcium-binding protein B
TBI: traumatic brain injury
UCH-L1: ubiquitin C-terminal hydrolase L1
